# Psychological Resilience as a Protective Factor for the Body Image in Post-Mastectomy Women with Breast Cancer

**DOI:** 10.3390/ijerph15061181

**Published:** 2018-06-05

**Authors:** Bernadetta Izydorczyk, Anna Kwapniewska, Sebastian Lizinczyk, Katarzyna Sitnik-Warchulska

**Affiliations:** 1 Institute of Applied Psychology, Faculty of Management and Social Communication, Jagiellonian University, 30-348 Krakow, Poland; kwapniewskaanna2@gmail.com (A.K.); k.sitnikwarchulska@gmail.com (K.S.-W.); 2 S.L.—Faculty of Psychology, SWPS University of Social Sciences and Humanities, 40-326 Katowice, Poland

**Keywords:** resilience, body image in post-mastectomy women with breast cancer

## Abstract

European statistics confirm a rise in breast cancer among contemporary women. Those suffering from cancer and undergoing a surgery (mastectomy) are undoubtedly considered to be in difficult situations. The range of the numerous negative and/or positive emotions, thoughts, and behaviours depend on many psychological factors such as psychological resilience. The authors are currently drawing a report on their own studies where they are trying to determine factors that protect body image resilience in women suffering from breast cancer after mastectomies. The research group consisted of 120 women after a short (up to 2 years) or a long (over 2 years) duration having elapsed since their mastectomy. The results of the research groups show that psychological resilience is a significant protecting factor for the body image that prevents the excessive development of negative self-esteem in post-mastectomy women. Female patients ought to be provided aid in the short time immediately after the procedure and afterwards, when they are less capable of tolerating negative emotions. In order to significantly improve the general body image resilience to emotional and cognitive distortions in post-mastectomy women who experienced breast cancer, it is recommended that psychological interventions (from psychoeducation to psychological assistance and specialist psychotherapy) are conducted systematically throughout the course of treatment.

## 1. Introduction

The research confirms a significant growth in breast cancer incidence among the contemporary female population [1]. One in eighteen women develops breast cancer before the age of eighty [2].

The review of the literature over the last 10 years shows the importance of the time that has elapsed since the mastectomy in the process of adaptation to life and the importance of mobilizing mental resources during recovery, including posttraumatic growth or the development of chronic stress and disorders [3,4,5,6,7,8,9]. In the case of breast cancer in a female, she can experience a state of deprivation of her basic emotional needs (particularly the need for a sense of security, a sense of having an impact on the course of one’s own life). This psychological stress is a significant element of the absence of mental wellbeing and exerts a negative impact on the course and the effectiveness of the patient’s treatment. The procedure of the mastectomy is perceived as a traumatic event resulting in a state of a psychological stress and often, also various mental disorders (anxiety and depressive symptoms), low self-esteem, and so forth [10,11,12,13]. In some studies, it has been noted that symptoms of arousal, avoidance, and intrusion similar to the characteristic of posttraumatic stress disorder (PTSD) may occur in post-mastectomy female patients [14,15]. Therefore, mastectomies can be considered a procedure that constitutes a potentially traumatic event, while cancer can be regarded as a potential source of psychological stress that hinders the psychosocial functioning due to the treatment and procedures it involves [16,17,18,19,20].

Analysis of the results of many contemporary studies indicates that sex and the time that has elapsed since the traumatic event (such as the surgery ending with the partial or total loss of the body’s organ) are significant criteria in differentiating forecasts in the recovery process, that is, the development of negative health effects or the development of posttraumatic growth [4,21]. Some studies suggest that these difficulties may subside and remain relatively stable after about two years. The elapsed time had a clear impact on the acceptance of the disease because the high level of this variable was present in women who had reconciled with the disease for up to 2 years after surgery [3,22]. 

A positive or negative adaptation after the mastectomy depends on various psychosocial factors. One of the most important variables is body image [23,24,25,26,27]. A review of the research conducted by Collins et al. [28] indicates that the dissatisfaction and poor well-being after a mastectomy are associated especially with body image changes, poor cosmesis, femininity, reoperations for acute and long term complications, and reconstructive problems. Breast cancer survivors who have lost a breast have had to cope with the tension and the discrepancy between the “self and body” and the social expectations of femininity [29]. Ramaseshan et al. [30] also indicated the relationship between body image and the difficulties in regulating emotions in the context of sex appeal in women after having a mastectomy.

On the one hand, some research findings suggest that post-mastectomy women, after breast reconstruction, valued the quality of their lives higher and felt more attractive than women who had undergone mastectomy without reconstruction. When a long time had elapsed since mastectomy, women put more focus on controlling their body weight by means of exercises and diet. This is probably related to the need of improving their self-image, including their body [31]. 

On the other hand, the results of a few studies confirm that the experienced of breast cancer and a mastectomy by a woman did not exclude the occurrence of posttraumatic growth (PTG) in this group of patients [8,32,33,34,35,36,37,38]. The phenomenon of posttraumatic growth may be related to a given individual’s psychological resources and psychological resilience, also with regard to body image. 

The theoretical assumptions and the developed model of variables refer to the concept of resilience, the body image resilience model developed by Choate [39] and contemporary cognitive and social (socio-cultural) body image formation models. Choate [39] notes that the awareness of socio-cultural pressures related to body weight and shape, as well as the capacity to cope with stress effectively, constitute as important factors that prevent the development of a dissatisfaction with the body, which is a significant factor that builds body image resilience in women. Self-esteem (thoughts and perception) and emotional satisfaction with the body are significant factors that contribute to the maintenance of a sense of a balanced body image and, thus, a restored health. Psychological resilience may constitute an individual’s predisposition to respond to the changing requirements of a given situation in a flexible manner, which is important in the process of coping with both traumatic and daily events [40,41]. Masten [42] claims that resilience pertains to phenomena in three areas: the accurate development despite putting the individual at great risk, the proper psychosocial functioning despite the experienced severe stress, and restoring balance or health after traumatic events (referred to as a ‘bounce-back’, that is, a capacity to rebound).

The meaning of psychological resilience and family resilience in determining the quality of life and psychological functioning of post-mastectomy women suffering from breast cancer has been mentioned by a few researchers [43,44,45,46,47,48,49]. The analysis conducted by Ristevska-Dimitrovska et al. [50] indicates that more resilient breast cancer patients have better body image, better future perspectives, and suffer from less severe adverse effects of cancer symptoms and therapy. Disturbances in the body image can be a barrier to resilience in patients with breast cancer [25,51]. Despite this point, the literature review has shown a lack of research studies examining the nature of the relationship between body image and resilience in post-mastectomy women with breast cancer. 

In building the research model, we were directed primarily by the need to examine the source material that has not been investigated and described thoroughly in a population of Polish women diagnosed with breast cancer. In the case of breast cancer, for female patients who had to be subjected to a mastectomy to save their lives and had lost a breast or both breasts, the quality of the maintained body resilience seems to have a great impact on the process of reclaiming their health and adapting to conditions of family life, professional life, and social life.

## 2. Research Objectives and Questions

Previous studies focus mainly on the search for risk factors of negative body image among women after mastectomies. For the purposes of the effective prevention and prophylaxis, the determination of protective factors may be particularly important. The effectiveness of treatment will depend on whether the women will develop a chronic psychological stress or whether or not they will experience the phenomenon of the so-called posttraumatic growth after a mastectomy. Thus, it can be pointed out that the little-known type of relationship between the body image and resilience among women after a mastectomy (total or partial), in the early and late phase after surgery, may have a potential impact on developing strategies to cope with traumatic events and thus, affect the effectiveness of further treatment of women with breast cancer.

The aim of the undertaken study was to determine the relationship between body image and resilience in women after a mastectomy. The specific objective is to determine the factors that affect body image resilience. Therefore, three research questions were formulated.

(1) Does psychological resilience explain the emotional, cognitive, and behavioural aspects of body image in post-mastectomy breast cancer female patients subjected to the study (and if so, to what extent)?
(2) Which of the aspects of psychological resilience verified in the study (the capacity to evoke positive emotions, that is, optimism and the capacity to mobilise oneself, openness to new experiences and humour) and/or the capacity to bounce back from negative experiences (consistency and determination in action, capacity to cope with negative emotions and to tolerate failures) explain the emotional, cognitive, and behavioural aspects of body image in post-mastectomy women?
(3) Is there a difference in the strength of correlation between the selected factors of psychological resilience provided in the second question and the emotional, cognitive, and behavioural aspects of the body image in breast cancer female patients subjected to a mastectomy up to two years and after two years before this study, and if so, what does it depend on?

## 3. Material and Methods

### 3.1. Procedure

This research was carried out in 2016–2017, after recruiting surveyed women from the patients of two metropolitan oncology hospitals in southern Poland (60 subjects) and from the participants of the Association Women after Mastectomy—“Amazonka” (clubs in the cities of southern Poland—60 subjects). Due to the goals of the research questions, the authors used, as a criterion for inclusion in the research group, the following condition: women who, at the time of the study, had completed a partial or total mastectomy due to diagnosed and treated breast cancer. The main criterion for exclusion from the study was the following: women who did not have a documented diagnosis of breast cancer with a mastectomy surgery performed at the same time. One hundred and thirty women aged 48–55 were qualified for the study. However, for further statistical analyses, 120 women were included as 10 women with malignant breast cancer and a history of mastectomy did not complete the study. Of the women who did not complete the study, there were six women had been hospitalized for a mastectomy (up to 2 years after a mastectomy) and four women who had undergone a mastectomy over 4 years ago from the time of the study (women from the “Amazons Club”). All women who did not qualify for further analysis did not complete the psychological tests for various reasons and did not complete the study (for example, due to deterioration of health during the test, or because they did not apply for the test).

### 3.2. Characteristics of the Study Group

Purposive sampling was used in selecting the study group participants. The criteria for selection to the clinical group included sex (female), breast cancer diagnosis, and completed mastectomy (partial or total, the latter involving the loss of both breasts). Each study participant had a medical history of treatment including hospitalization experienced before the study due to breast cancer and a mastectomy (either partial or total). As many as 120 women diagnosed with breast cancer specified in their medical records and who had completed a mastectomy were included in the study in total. All of them were at a similar age (ranging from 48 to 55 years old, M = 54.0). The largest group of respondents were women over 50 years old, which is consistent with the epidemiological data from previous studies that shows an increase in breast cancer incidence in that age range [52]. 

The results suggested that patients with breast cancer are most concerned about the body image in the immediate postoperative period and problems with the body image decrease after about two years [53,54]. The specificity of the course phases of mourning and the time needed to experience psychological changes associated with the loss of time requires the adaptation (as reported in the literature of at least one to two years) to the new reality after a mastectomy.

Considering the above factors, the research group (study cohort) was divided into two subgroup. In order to make the analysed results clearer, the group consisting of post-mastectomy women, up to 2 years after a mastectomy, is referred to as clinical group 1, whereas the group of women who had undergone mastectomy over 2 years before the study is called clinical group 2. It was recognized according to the literature that group 1 (up to 2 years after a mastectomy) are women experiencing potential psychological processes related to the dynamics of mourning phases whereas group 2 women (two years after a mastectomy) are in the phase of adapting to the new reality in everyday life.

### 3.3. Organisation and Course of the Study

In the research model, two major variables were determined. The first of the said variables was referred to as psychological resilience (independent variable). It is a variable defined as a set of certain psychological competencies with regard to the effective, flexible coping in severely stressful situations, manifested as a capacity to evoke positive emotions (optimism and capacity to mobilise oneself, openness to new experiences and humour) and as a capacity to bounce back from negative experiences (consistency and determination in action, capacity to cope with negative emotions and to tolerate failures) [55,56]. 

Body image, the dependent variable, is describing the following constituents [57]:

(1) Dissatisfaction with the body—a variable describing the level of negative emotions towards one’s own body and the level of discomfort experienced with regard to the body and appearance.
(2) Control over the body—a variable describing the level of difficulty in identifying one’s own physical and emotional states related to the feelings of anxiety and the level of difficulty in controlling and experiencing the bond with one’s own body.
(3) Perception of the body—a variable describing the tendency to perceive one’s body size and individual body parts negatively.
(4) Self-assessment of the body—a variable describing the level of general acceptance and self-assessment of one’s own body, shape, appearance, and weight.
(5) Intimate relationships—a variable describing the degree of emotional and bodily satisfaction with physical (intimate) contact with another person.
(6) Weight control and attitude towards eating—a variable describing the level of control over behaviour related to body weight and an excessive focus on eating.
(7) Physical attractiveness—a variable describing an individual’s behaviour related to manifesting her femininity by means of clothing, makeup, and other behaviour aimed to enhance the attractiveness of the body, and so forth.

### 3.4. Instruments

The predictor variable (psychological resilience) and its components, were measured using the Resilience Measurement Scale (SPP-25, Psychology Institute, University of Lodz, Poland) developed by Ogińska-Bulik and Juczyński [58]. The scale consists of a General psychological resilience subscale (a summary result for all the subscales of SPP-25—GenR), the subscales of SPP-25, describing the capacity to evoke positive emotions (Optimism and capacity to mobilise oneself—OCM, Openness to new experiences and humour—ONEH), and subscales describing the capacity to bounce back from difficult situations (consistency and determination—CD, coping with negative emotions—CNE, failure tolerance—FT). The Resilience Measurement Scale (SPP-25) is characterised by satisfactory psychometric properties (the Cronbach alpha for the entire scale was estimated at 0.89, while for individual factors, the Cronbach alpha ranged from 0.67 to 0.75). Test-retest reliability measured after 4 weeks reached 0.85, meaning that the resilience was highly stable [58].

Dissatisfaction with the body, control over the body, and the perception of the body were measured using the Polish version of the Body Attitude Test developed by Brytek-Matera and Probst [59]. Whereas self-assessment of the body, intimate relations, physical activity, eating attitude, and weight control were measured using the Body-Self Questionnaire developed by Mirucka [60]. The Body-Attitude Test (BAT) in the Polish version is characterised by satisfactory psychometric properties as well as reliability. The Cronbach alpha for the entire scale ranged from 0.85 to 0.90 [59]. The higher the score, the worse the self-perception of one’s body. The BAT questionnaire includes 3 subscales (general dissatisfaction; lack of familiarity with the body; negative appreciation of body size). Likewise, the Body Self Questionnaire developed by Mirucka [60] has satisfactory psychometric properties (Cronbach alpha for the entire scale estimated at 0.93, ranging from 0.74 to 0.89 for individual subscales). It is a method that comprises of 41 questions assessed on a 7-item sale from 0—completely disagree to 6—completely agree or 6—completely disagree to 0—completely agree, in the case of reverse questions. 

### 3.5. Ethical Approval

Ethical approval was obtained from relevant institutional ethical review committees and the research conducted in accordance with national and international regulations and guidelines. The written informed consent was obtained from all participants. (Research ethics committee of institute of applied psychology Jagiellonian University, December 2016).

### 3.6. Statistical Methods

The study results were statistically analysed using the IBM Statistical Package for the Social Sciences (SPSS) Statistics software (IBM Corp. Released 2017. IBM SPSS Statistics for Windows, Version 25.0. IBM Corp., Armonk, NY, USA). Due to the absence of the normal distribution for the analysed variables, differences between the groups were measured using the non-parametric Mann-Whitney *U* test for both groups. First, a comparison of clinical group 1 and clinical group 2 was conducted. In the statistical analysis of the strength of prediction between the variables “psychological resilience” and “body image resilience”, a stepwise linear regression model was applied. As an acceptance or rejection criterion for the proposed hypotheses, the level of significance was set at *p* = 0.05. 

## 4. Results

In the first stage of analysis of the study results, the research group was characterized by the description of sociodemographic variables (residence environment, education, marital status), and variables describing the character of the mastectomy surgery (partial or total), time of mastectomy (up to 2 years and more than 2 years), and having/not having had breast reconstruction. These data are presented in Table 1 and Table 2. The selected distributions of variables in the population of women after a mastectomy are presented for the selected sociodemographic variables.

The largest number of respondents lived in the urban environment (61.60%). The most represented women in the study population are married women (62.5%) and women with secondary and higher education (82.5%).

In the next stage of statistical and clinical analysis of the results, the following characteristics of variables were taken: the body image after a mastectomy in women with breast cancer up to two years or more than two years after a mastectomy.

### 4.1. Characteristics of the Body Image in Post-Mastectomy Women Suffering from Breast Cancer Up to Two Years or over Two Years after the Mastectomy

In the description of the measurement of the level of indicators of both independent and dependent variables, a reference was made to central tendency measures (mean values and standard deviations). Table 3 presents the study results concerning the comparative analysis regarding mean values of indicators of the “body image resilience” variable for women, up to 2 years after a mastectomy, and for women, over 2 years after a mastectomy. 

As for a comparison between the mean intensity of the constituents of the ‘body image resilience’ variable in clinical groups 1 and 2, the statistical analysis revealed no significant differences between the aforementioned groups. The results confirmed that the post-mastectomy female participants manifested a similar level of cognitive, emotional, and behavioural components of the body image regardless of the time elapsed since the procedure.

All the verified emotional constituents describing the body image, from general dissatisfaction with the body manifested by the respondents, the lack of control over sensations of their bodies, the identification of their own physical and emotional states, to the experienced level of emotional and bodily satisfaction with physical (intimate) contact with another person proved similar in all the study participants regardless of the time elapsed since the mastectomy (up to two years and over two years). The mean values obtained for the body image describing the dissatisfaction with the body, perception and control, as well as indicators of caring for one’s physical attractiveness remained within the average score. In turn, the general self-acceptance of the body (the level of general acceptance of the body and the level of experienced emotional and bodily satisfaction with physical (intimate) contact with another person, as well as the tendency to control body-weight related behaviour and an excessive focus on eating proved to be slightly higher than the emotional dissatisfaction with the body and control over bodily sensations. It was only in the case of the ‘weight control and eating attitudes’ variable that a notable difference was observed between the female participants who had undergone a mastectomy over 2 years prior to the study and those who had been subjected to this procedure up to 2 years prior to the study. The latter group more often reported behaviour characterised by excessive control over eating and body weight. Though the said difference did not prove statistically significant, a distinct trend could be observed due to the obtained *p*-value (Table 1).

### 4.2. Characteristics of Psychological Resilience in Women Who Underwent a Mastectomy Up to Two Years or over Two Years before the Study

As for a comparison between the mean values of the constituents of the “psychological resilience” dependent variable in the female participants up to two years (clinical group 1) and over two years (clinical group 2) elapsed since the mastectomy, the statistical analysis revealed significant differences between the said groups (Table 4).

In the course of a comparative analysis of the means, the results indicate the presence of significant differences between women, up to 2 years after a mastectomy (clinical group 1) and women, over 2 years after a mastectomy (clinical group 2) in general psychological resilience, which proved to be significantly higher in clinical group 1. The female participants in clinical group 1 obtained mean score of general psychological resilience at the upper threshold of the average score, whereas the women from clinical group 2 scored averagely at the lower threshold of the average score. The average score in all the described factors for the capacity to bounce back from difficult situations (consistency and determination, coping with negative emotions and failure tolerance), as well as scores for the capacity to evoke positive emotions, was rather higher in clinical group 1. However, significant differences between the participants of the two groups pertained solely to the general psychological resilience and one constituent of psychological resilience, namely, the capacity to bounce back from difficult situations. In this regard, a significantly higher score was obtained by the women who had undergone mastectomy up to 2 years before the study presented in this article. As for the capacity to evoke positive emotions, no significant differences were observed between the two groups. 

### 4.3. Psychological Resilience as a Protective Factor for Body Image in Post-Mastectomy Women

The obtained research materials also allowed the authors to verify the question “to what extent can psychological resilience in post-mastectomy women serve as a protective factor or a risk factor for body image disorders (in regard to emotions, observations, thoughts related to the body and body-oriented behaviours)?” Specifically, which of the examined factors of psychological resilience, that is, the capacity to evoke positive emotions (optimism and capacity to mobilise oneself, openness to new experiences and humour) and/or the capacity to bounce back from negative experiences (consistency and determination in action, capacity to cope with negative emotions and failure tolerance) are significantly correlated with emotional, cognitive, and behavioural aspects of the body image in post-mastectomy women?

In order to answer the above research question, the next step of the statistical analysis involved subjecting the findings to a stepwise regression analysis. An attempt was made to estimate the strength and the direction of the possible predictors of the body image. Due to the large number of independent variables compared to the number of the study participants (*n* = 120), a linear regression with backward stepwise selection was used). Subsequent steps in the model eliminated variables with poor coefficients excessively correlated with other variables. Hence, redundant and poor predictors were eliminated from the model, producing only models with a satisfactory prediction [61]. Table 5 presents only the significant stepwise regression models of the investigated area of variables. The data on the coefficients of eliminated statistically eliminated variables were not included in the description. 

The above table determines the significant predictors for the “body image resilience” dependent variable. A regression analysis was conducted in the entire study group (clinical groups 1 and 2) and for each of the said clinical groups individually. This allowed the authors to identify the role and the meaning of various predictors depending on the time elapsed since the mastectomy. 

Based on the obtained adjusted R^2^, it can be concluded that the level of the explained variation of the “body image resilience” dependent variable ranged from 6% to 25%. Although nearly all the regression models proved to be statistically significant (*F*-test), the R^2^ values clearly suggest that the dependent variable can also be affected by other factors that were not taken into account in this study. The results of the statistical analysis shown in Table 3 present factors of psychological resilience that are significantly correlated with the emotional, cognitive, and behavioural aspects describing the specificity of the body image in post-mastectomy women with due account given to the measurement of the strength of the correlation between the variables with regard to the overall study group and the individual clinical groups (women, up to two years after a mastectomy and over two years after a mastectomy).

The stepwise regression analysis proved that the score obtained for general psychological resilience (defined as a general disposition of an entity, describing the capacity of the study participants to bounce back from difficult situations and negative emotions, and the capacity to evoke positive emotions) was a significant independent variable of factors constituting body image resilience in post-mastectomy women. Considering the time elapsed since the mastectomy for the study participants until the moment of the study and the results of the conducted regression analysis, we can identify a number of various component predictors of psychological resilience. Moreover, we can notice the significance of the time due to which various types of predictors can be distinguished for the very same variable. The statistical analysis proved that the variables constituting body image resilience are correlated in particular clinical groups by different predictors in different ways. 

The predictor of the level of self-acceptance of the body differs depending on the clinical group. Within the entire study group (both clinical group 1 and clinical group 2), this role is played by general psychological resilience; whereas it is played by optimism and capacity to mobilise oneself in clinical group 1, and coping with negative emotions in clinical group 2. All these predictors indicate a considerable positive strength of influence. This high variability in the significant predictors suggests that, in this case, the time elapsed since the mastectomy is significant;

- In both clinical groups, openness to new experiences acts as the predictor. Beta coefficients (from 0.426 to 0.568) indicate a positive influence of the predictor;
- The predictor of the level of control over weight and eating attitude differs depending on the clinical group. Within the overall study group (both clinical group 1 and clinical group 2) this role is played by failure tolerance; it is played by failure tolerance and consistency and determination in clinical group 1, whereas, in clinical group 2, it is played by coping with negative emotions. Apart from the “consistency and determination” variable, all predictors indicate a positive influence on the dependent variable;
- The predictor of physical attractiveness in the entire study group is general psychological resilience. In clinical group 1, no significant predictor for this variable was identified, whereas, in clinical group 2, this role was played by consistency and determination. The mentioned predictors have a strong positive influence on the dependent variable;
- The predictor of the level of control over the body within all the examined groups of women is general psychological resilience. The beta coefficients (from −0.568 to −0.468) indicate a negative influence of the predictor on the dependent variable;
- The predictor of the level of perception of the body in the entire study group and in clinical group 2 was coping with negative emotions, while in clinical group 1, this role was played by failure tolerance. All predictors indicate a negative strength of the influence. The greatest influence was identified for clinical group 2;
- The predictor of the level of dissatisfaction with the body in all the examined groups of women is general psychological resilience. The beta coefficients (from −0.509 to −0.413) indicate a negative influence of the predictor;

The remaining constituents of the “psychological resilience” variable (included in the research model) are not to be significantly correlated with the constituents of the body image in the post-mastectomy female participants. It is possible to hypothesize that the remaining variables that did not show the correlation may have an impact, although considering the weakness of the research tools, it is difficult to rule out such an impact. It seems to be an important issue to undertake in further research.

## 5. Discussion

The statistical analysis of the findings proved that only some of the constituents of the psychological resilience and body image variables remained significantly correlated. The obtained results have pointed to the existing significant positive relationship between general resilience and body image in post-mastectomy women with breast cancer. The higher level of resilience leads to better self-esteem and satisfaction with the image of one’s body. Other authors also indicate a possibility of similar relationship in their research [25,50] or models [51]. 

The analysis of the collected research material confirmed that post-mastectomy women differ with respect to the level of experienced psychological resilience depending on the time elapsed since the procedure. Some researchers point to the importance of the criterion of time that has elapsed since mastectomy in the process of adapting to life and the strength of mobilization of mental resources during recovery [3,4,5,6,7,9]. It is worth mentioning that there is a lack of research analyzing the relationship between resilience and body image, including the post-mastectomy time criterion. The results of the statistical analysis in our study suggest higher psychological resilience and greater capacity to bounce back from difficult situations in a short period of time elapsed since the mastectomy. This seems consistent with the research on posttraumatic growth (PTG), which can be affected by psychological resilience indirectly, as mentioned above. In their discussion on the concept of posttraumatic growth, Tedeschi and Calhoun [61,62], Manne et al. [63], Lechner et al. [64], and Semmer et al. [45] noted that the highest number of positive changes occur in the early stages of cancer (for example, from two weeks to two months following a difficult experience). As in the research by Ogińska-Bulik [65], in the study presented in this article, the body image proved to be less significantly explained by the capacity to evoke positive emotions The high capacity to develop the ability to bounce back from difficult situations in post-mastectomy patients was also noted in studies by Krok and Kubiec [66] and Semmer [45]. It is possible that accepting a loss of one or both breasts requires a greater capacity to accept, tolerate, and cope with the negative emotions that occur in such a situation than evoking positive emotions or humour.

It is also possible that, in the women experiencing loss and pervasive deformity of the body (the loss of one or both breasts), both defence and adaptive mechanisms become less effective over time, causing difficulties in coping with one’s daily life and psychosocial needs. According to the findings, the shorter the time elapsed since the procedure and the stage of experienced loss of part of the body, the more promotion towards defence mechanisms. In the first stage after mastectomy, a woman can develop more positive visions of treatment and rehabilitation that give hope for the future (restored health). Undoubtedly, hope is an important psychological variable in the treatment process. A high level of hope for implementing one’s goals (success) promotes engagement and constructive strategies for coping with cancer [22,67]. The psychosocial response in women after having a longer time elapse since their mastectomy may be characterised by growing emotional instability, a sense of lost physical attractiveness, compromised self-esteem, a lack of control over one’s own life, as well as symptoms of depression and anxiety.

Importantly, in the investigated group of post-mastectomy patients, women who did not undergo breast reconstruction prevailed. This fact could affect the study results. Studies by other authors suggest that the quality of life and the feeling of attractiveness and femininity among women who underwent breast reconstruction improved compared to that reported before reconstruction [31].

This variable explains self-assessment and dissatisfaction with the body to the greatest degree. In the presented original study, post-mastectomy women characterised by high psychological resilience manifested a higher level of self-acceptance of the body and a lower dissatisfaction with the body, which is partially confirmed by studies conducted by McGrath et al. [68], who proved psychological resilience to be negatively correlated with the dissatisfaction with the body in young female college students, as confirmed in the study presented in this article. 

High psychological resilience is also connected to a higher probability of posttraumatic growth [69,70,71]. Psychological resilience is related to greater openness and a more active seeking of solutions, more effective coping strategies employed in difficult situations [65,69,70,72], as well as higher resilience to social pressure regarding appearance [39,73]. It can also refer to women after mastectomy. Greater self-acceptance of the body and lower dissatisfaction with the body among women with higher resilience may indicate their ability to positively re-evaluate the experienced mutilation or to cognitively reinterpret it as a symbol of strength and overcome difficulties. 

Based on the analysis of the study results, it is possible to identify the meaning of ongoing psychoeducation conducted by medical staff (physicians, psychologists, nurses) from the very beginning of treatment in breast cancer participants, oriented at teaching constructive strategies for coping with stress and negative emotions (particularly anger, fear, sadness), and for preventing negative thinking about the future.

Due to the possibility of the event of a predicted further reduction in the psychological resilience (as confirmed by the study results) after a mastectomy, it is worth seeking professional help at the initial stage after a mastectomy. Professional psychological assistance could be focused on educating patients on the building of constructive and positive emotional patterns to respond to difficult situations, particularly based on further developing the capacity to distance oneself from such situations and to evoke positive emotions. 

### Limitations and Future Directions

Nonetheless, the experiments presented in this article are not exempt from some limitations, which suggests that care should be taken when generalizing the obtained findings on a wide population of breast cancer patients. The aforementioned care is also justified by limitations arising from a relatively low number of participants in the study group who differed in terms of age and disease duration. When exploring this research area further, one should give due account to longer-term longitudinal studies on a larger group of post-mastectomy female patients that would be more representative of the Polish population. Moreover, the studies can be also be limited to some extent by tools used for measuring variables (despite a well-justified need for applying such tools) based primarily on self-assessment. Although the research methods do not eliminate the acceptance of the declarative nature of the answer, the functioning of women in the process of coping with the traumatic event of cancer might make it difficult to obtain the data required for the study.

There are undoubtedly more protective factors that influence the mental functioning of breast cancer female patients with a positive effect on treatment than the variables verified in this study. The criterion of breast reconstruction or non-mastectomy is a factor that is worth considering in future studies. However, as suggested in the literature, psychological resilience is a psychological variable with a significant impact on either the positive or negative course of treatment in individuals suffering from chronic psychosomatic diseases.

According to the authors of this paper, the above-presented limitations do not negate the novelty of the obtained research data on the significance of psychological resilience in the functioning of the body image of women suffering from breast cancer who had undergone mastectomies.

## 6. Conclusions

The analysis of the obtained results provided the answer to the posed research questions. The said results serve as a basis for the adoption of the general conclusions specified below.

(1) Psychological resilience is a significant protecting factor for the body image that prevents the excessive development of negative self-esteem in post-mastectomy women.
(2) For all the post-mastectomy female participants who suffered from breast cancer, the general psychological resilience proved to be a significant protecting factor of body image.
(3) Importantly, female patients ought to be provided aid in the period immediately after the procedure and afterwards when they are less capable of tolerating negative emotions.
(4) In order to significantly improve the general body image resilience to emotional and cognitive distortions in post-mastectomy women who experienced breast cancer, it is recommended that psychological interventions (from psychoeducation to psychological assistance and specialist psychotherapy) are conducted systematically throughout the course of the treatment.

## Figures and Tables

**Table 1:** The characteristics of women from the clinical group (sociodemographic variables) (*n* = 120).

Sociodemographic Variables		Research Group *n* = 120
Age		M = 54.0
Place of residence	Place up to 50 thousand residents	14.2%
	Town 50–100 thousand residents	24.2%
	City 100–200 thousand residents	33.3%
	City over 200 thousand residents	28.3%
Marital status	Currently not in a relationship	10.0%
	Married	62.5%
	In a partnership	1.7%
	Divorced/separated	5.8%
	Widow	20.0%
Education	Junior high school or elementary school	7.5%
	Basic vocational school	10.0%
	High school (college, post-college)	45.3%
	University	37.2%

**Table 2:** The characteristics of women in the research group (data related to mastectomy surgery).

Data on Previous Treatment		Research Group *n* = 120
Breast prosthesis	Having a breast prosthesis	59.2%
	Lack of breast prosthesis	40.8%
Breast reconstruction	After breast reconstruction	5.0%
	Without breast reconstruction	95%
Time from surgery	Less than two years	53.3%
	Over two years	46.7%
Type of treatment	Complete mastectomy	64.2%
	Partial mastectomy	35.8%

**Table 3:** The comparison of raw results (Mann-Whitney *U* test) regarding indicators of the dependent variable ‘body image’ between clinical group 1, and (clinical group 2).

Body Image	Women, up to 2 Years after Mastectomy (Clinical Group 1 *n* = 64)		Women, over 2 Years after Mastectomy (Clinical Group 2 *n* = 56)		*U*	*p*
	M	SD	M	SD		
Dissatisfaction with the body	6.16	3.847	6.54	3.799	1662.00	0.492
Control over the body	10.02	4.709	10.04	5.253	1703.00	0.639
Perception of the body	10.31	6.976	10.20	6.746	1775.00	0.929
Self-assessment of the body	50.42	13.836	49.11	15.165	1674.00	0.535
Intimate relationships	48.23	11.447	46.07	14.720	1642.50	0.431
Weight control and eating attitudes	36.44	11.042	33.96	8.926	1435.00	0.060
Physical attractiveness	19.25	5.866	19.45	6.755	1748.00	0.817

Legend: Emotional factors: dissatisfaction with the body—negative emotions oriented towards one’s own body; Control over the body—the level of difficulty in identifying one’s own physical and emotional states related to the feeling of anxiety and the level of difficulty in controlling one’s own body and feeling a bond with one’s own body; Intimate relationships—the degree of emotional and bodily satisfaction with physical (intimate) contact with another person. Cognitive factors: perception of the body—a tendency to perceive one’s body size and its individual parts negatively; Self-acceptance of the body—the level of general acceptance and self-assessment of the body, its size, shape, and weight. Behavioural factors: control of weight and eating attitudes—the level of control over body-weight related behaviours and excessive focus on eating; Physical attractiveness—an individual’s behaviours related to the manifestation of her femininity by means of clothes, makeup, and other behaviours aimed to improve the attractiveness of the body, and so forth.

**Table 4:** The comparison of raw results (Mann-Whitney *U* test) regarding indicators of the “psychological resilience” independent variable between clinical group 1, and clinical group 2.

Psychological Resilience	Women, up to 2 Years after Mastectomy (Clinical Group 1 *n* = 64)		Women, over 2 Years after Mastectomy (Clinical Group 2 *n* = 56)		*U*	*p*
	M	SD	M	SD		
General psychological resilience	73.33	13.073	67.89	14.488	1383.00	0.031
Capacity to evoke positive emotions						
Optimism and capacity to mobilise oneself	13.00	3.281	12.48	3.653	1621.00	0.366
Openness to new experiences and humour	15.70	3.001	14.80	2.920	1475.50	0.094
Capacity to bounce back from difficult situations						
Consistency and determination	15.91	2.799	14.63	3.102	1371.00	0.026
Coping with negative emotions	13.95	3.174	12.43	3.515	1310.00	0.011
Failure tolerance	14.77	2.810	13.55	3.308	1386.00	0.032

Legend: General psychological resilience—a set of psychological competencies in effective and flexible coping in severely stressful situations described by the means of a sum of raw scores from the subscales of the Resilience Measurement Scale (SPP-25) [58]; Capacity to evoke positive emotions described by optimism and capacity to mobilise oneself, and the level of openness to new experiences and humour levels); Capacity to bounce back from difficult situations described by the level of consistency and determination in action, the level of capacity to cope with negative emotions and the level of failure tolerance.

**Table 5:** The summary of significant stepwise regression models for the ‘body image resilience’ dependent variable in the group of post-mastectomy women (*n* = 120); clinical group 1 and clinical group 2.

Dependent Variable: Constituents of the Body Image	Post-Mastectomy Women (for Clarity in the Table Are Only Significant Predictors)		
	All Participants in Total	Up to 2 Years after Mastectomy	Over 2 Years after Mastectomy
Self-acceptance of the body	GenR 0.540 ***	OCM 0.504 ***	CNE 0.609 ***
Intimate relationships	ONEH 0.426 ***	ONEH 0.568 ***	ONEH 0.449 ***
Weight control and eating attitudes	FT 0.290 ***	FT 0.488 ** CD −0.361 *	CNE 0.375 **
Physical attractiveness	GenR 0.533 ***	ns	CD 0.435 ***
Control over the body	GenR −0.510 ***	GenR −0.468 ***	GenR −0.568 ***
Perception of the body	CNE −0.267 **	FT −0.282 *	CNE −0.512 *
Dissatisfaction with the body	GenR −0.440 ***	GenR −0.413 ***	GenR −0.509 ***

*** *p* < 0.001; ** *p* < 0.01; * *p* < 0.05; ns—non-significant. Legend: Psychological resilience: OCM—Optimism and capacity to mobilise oneself; ONEH—Openness to new experiences and humour; CD—Consistency and determination; CNE—Coping with negative emotions; FT—Failure tolerance; GenR—General psychological resilience. Body image: dissatisfaction with the body, that is, negative emotions oriented towards one’s own body; Control over the body—the level of difficulty in identifying one’s own physical and emotional states related to the feeling of anxiety and the level of difficulty in controlling one’s own body and feeling a bond with one’s own body; Intimate relationships—the degree of emotional and bodily satisfaction with physical (intimate) contact with another person. Cognitive factors: perception of the body (a tendency to perceive one’s body size and its individual parts negatively); Self-acceptance of the body—the level of general acceptance and self-assessment of the body, its size, shape, and weight. Behavioural factors: control of weight and eating attitudes (the level of control over body-weight related behaviours and excessive focus on eating); Physical attractiveness—an individual’s behaviours related to manifesting her femininity by means of clothes, makeup and other behaviours aimed to improve the attractiveness of the body, and so forth.
